# Predicting remission after internet-delivered psychotherapy in patients with depression using machine learning and multi-modal data

**DOI:** 10.1038/s41398-022-02133-3

**Published:** 2022-09-01

**Authors:** John Wallert, Julia Boberg, Viktor Kaldo, David Mataix-Cols, Oskar Flygare, James J. Crowley, Matthew Halvorsen, Fehmi Ben Abdesslem, Magnus Boman, Evelyn Andersson, Nils Hentati Isacsson, Ekaterina Ivanova, Christian Rück

**Affiliations:** 1 Centre for Psychiatry Research, Department of Clinical Neuroscience, Karolinska Institutet, & Stockholm HealthCare Services, Region Stockholm, Huddinge, Sweden; 2 Department of Psychology, Faculty of Health and Life Sciences, Linnaeus University, Växjö, Sweden; 3 CAP Research Centre, Stockholm Health Care Services, Region Stockholm, Stockholm, Sweden; 4 Department of Genetics, University of North Carolina at Chapel Hill, Chapel Hill, USA; 5 Research Institutes of Sweden, Kista, Sweden & Royal Institute of Technology, Stockholm, Sweden; 6 Department of Learning, Informatics, Management and Ethics, Karolinska Institutet, Solna, Sweden

**Keywords:** Depression, Medical genetics

## Abstract

This study applied supervised machine learning with multi-modal data to predict remission of major depressive disorder (MDD) after psychotherapy. Genotyped adult patients (*n* = 894, 65.5% women, age 18–75 years) diagnosed with mild-to-moderate MDD and treated with guided Internet-based Cognitive Behaviour Therapy (ICBT) at the Internet Psychiatry Clinic in Stockholm were included (2008–2016). Predictor types were demographic, clinical, process (e.g., time to complete online questionnaires), and genetic (polygenic risk scores). Outcome was remission status post ICBT (cut-off ≤10 on MADRS-S). Data were split into train (60%) and validation (40%) given ICBT start date. Predictor selection employed human expertise followed by recursive feature elimination. Model derivation was internally validated through cross-validation. The final random forest model was externally validated against a (i) null, (ii) logit, (iii) XGBoost, and (iv) blended meta-ensemble model on the hold-out validation set. Feature selection retained 45 predictors representing all four predictor types. With unseen validation data, the final random forest model proved reasonably accurate at classifying post ICBT remission (Accuracy 0.656 [0.604, 0.705], P vs null model = 0.004; AUC 0.687 [0.631, 0.743]), slightly better vs logit (bootstrap D = 1.730, *P* = 0.084) but not vs XGBoost (D = 0.463, *P* = 0.643). Transparency analysis showed model usage of all predictor types at both the group and individual patient level. A new, multi-modal classifier for predicting MDD remission status after ICBT treatment in routine psychiatric care was derived and empirically validated. The multi-modal approach to predicting remission may inform tailored treatment, and deserves further investigation to attain clinical usefulness.

## Introduction

Major Depressive Disorder (MDD) is a leading cause of disability affecting >260 million individuals worldwide [1]. In Europe and US alone, around 35 million are estimated to suffer from untreated MDD with a treatment gap of approximately 50% [2, 3]. Treatment of MDD includes psychotropic medication, psychotherapy, and their combination. Adequate treatment accessibility for MDD would produce an estimated net benefit of ~230 billion USD in productivity gains worldwide [4]. For mild or moderate MDD, cognitive behavioural therapy (CBT) and its more cost-effective online version (ICBT) are empirically supported [3, 5] and recommended by guidelines [6, 7]. However, a substantial portion of individuals—estimates range from 10 to 60%—do not respond sufficiently to ICBT [8, 9]. As there is room for improvement, researchers have begun to investigate what variables predict symptom reduction [3, 8, 10], remission status [11], and other outcomes proximal to ICBT response such as adherence [12], and dropout [13]. Identifying replicable predictors could inform clinical decision-making allowing for better tailored intervention and care for these patients.

Depression is a polygenic condition [14] also influenced by environmental factors and gene-environment interactions [15]. Consequently, CBT response is likely to be a multi-factorial trait for which a range of predictors have been identified, including prior psychological treatment [16], baseline symptom severity [8, 10, 16, 17], time-updated weekly symptom severity [18], disability status [10], quality of life, computer comfortability [19], education [10], and sex [8]. Also, process-specific ICBT predictors [12], and Polygenic Risk Scores (PRS) [20] have been suggested, although associations between PRSs and CBT outcomes reported so far are weak [17]. A common characteristic of predictive modelling studies of psychotherapy outcomes is that prediction performance has room for improvement, suggesting larger sample sizes and a richer multi-modal (combined data of different types) predictor approach [21]. For leveraging such high-dimensional and complex data for prediction, standard linear regression modelling is suboptimal as it requires manual specification of interactions and non-linearities and linear models consequently tend to be underfitted to such data. To strengthen model derivation and performance, while deliberately sacrificing some model transparency, flexible non-linear algorithms can be fitted that automatically handles complex patterns in data for optimised model predictions. With proper control for overfitting and validation on unseen hold-out data, we can empirically test accuracy and generalisability of flexible model predictions to new patients. Although the application of such machine learning methodology for ICBT outcome prediction has insofar been rare, the field is progressing fast [21–24].

The present study investigated a multi-modal data-driven approach to predict post ICBT remission status under ecologically valid conditions using a sample of patients with mild-to-moderate MDD treated with ICBT at the Internet Psychiatry Clinic (IPSY) in Sweden. Critically, all patients had time-stamped online behaviour registered in the digital treatment platform and had been genotyped. This enabled inclusion of a wide range of pre-treatment predictors, including demographic and clinical variables, process variables (e.g., time for a patient to complete a baseline questionnaire), and PRSs for different potentially predictive traits (e.g., PRS for MDD other psychiatric disorders). Both well-known binomial logistic regression and two more flexible non-linear algorithms were applied in a machine learning pipeline of cross-validation (CV) resampled predictor selection and model derivation followed by temporal external validation on the most recently treated patients for predicting post ICBT remission in MDD.

## Materials and methods

### Ethical considerations

All participants provided written informed consent, the study adheres to the Declaration of Helsinki and was ethically approved by the Regional Ethics Board in Stockholm (dnr 2009/1089-31/2 & 2014/1897-31).

### Patients and setting

Details on patient referral, recruitment, treatment, and study setting have been reported elsewhere [20]. In summary, 894 patients (≥18 yrs) with mild-to-moderate MDD undergoing a standardized therapist-supported ICBT protocol were recruited from the Internet Psychiatry Clinic (IPSY) in Stockholm from 2008 through 2016. IPSY is the largest ICBT unit in Sweden. Participants donated blood for genotyping and much of their ICBT process activity on the online platform was logged (e.g., time of day and duration of questionnaire completion). The procedure encompassed online screening, on-site psychiatric assessment, initial exclusion/referral (if unable to read or write Swedish, severe MDD, moderate/high suicide risk, recent medication changes, bipolar or psychotic disorder, drug dependence). Treatment with ICBT was for 12 weeks in sequential homework assignment format, with first follow-up at post measurement at treatment completion and second follow-up 3–6 months after treatment start [25].

### Outcome

The primary outcome was the Montgomery-Åsberg Depression Rating Scale-Self report (MADRS-S) completed at post treatment classifying a total score ≤10 as remission, and >10 as no remission. The MADRS-S is a widely used instrument for assessing MDD [26, 27]. While somewhat varying cut-offs have been suggested for the MADRS-S across studies [28], we settled on the clinician-rated version (MADRS) cut-off [29]. In an additional sensitivity analysis, the alternative cut-off of ≤12 as remission, and >12 as no remission was used to assess our prediction model robustness to slight variation in symptom severity for classifying post-treatment remission.

### Modelling

**Fig. 1:**
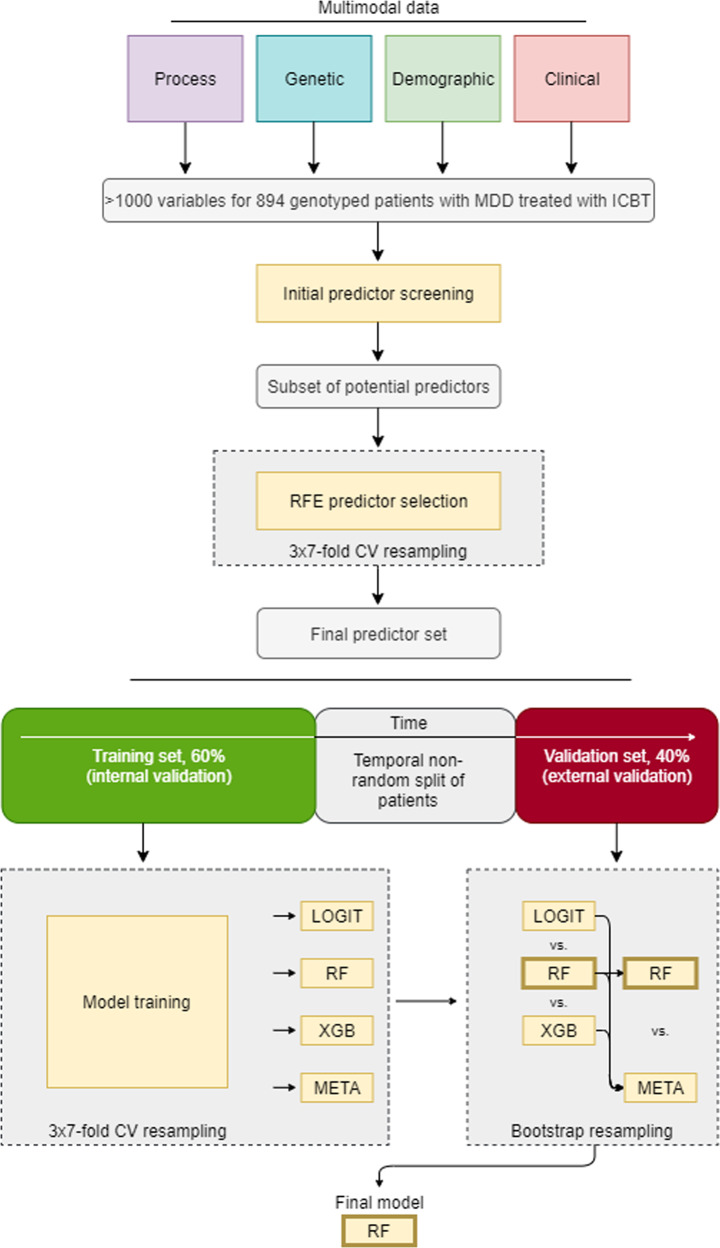
Workflow of predictor selection, model derivation, and validation. *CV* cross-validation, *ICBT*, Internet-based Cognitive Behaviour Therapy, *LOGIT* binomial logistic regression, *MDD* Major Depressive Disorder, *RF* random forest, *RFE* recursive feature elimination, *XGB* eXtreme gradient boosted trees, *META* blended meta-ensemble model of LOGIT, RF, and XBG.

Modelling involved several steps. See Fig. 1 for a modelling flowchart.

### Predictor selection

Only variables available at pre-treatment baseline were allowed as predictors. For predictive modelling, similar predictors (e.g., MADRS-S completed at screening and MADRS-S completed at pre-treatment start) are allowed as potential predictors in the same model. See [30, 31] for the distinction and trade-off between optimising a predictive model (model fit is paramount) versus optimising an explanatory model (unbiasedness and precision of model coefficients are paramount).

Given the high dimensionality of the initial dataset (>1000 variables), psychological and psychiatric expertise (JW, JB, CR) was applied to screen out a subset of potential predictors of unmanageable detail (e.g., item level responses on psychometric scales), limited utility (e.g., genetic sex), or measured past baseline (e.g., weekly symptom ratings during ongoing treatment). Statistical screening of predictor suitability was also applied (near-zero variance predictor cut-off: ratio 95/5 for 1^st^/2^nd^ most common categorical values or 10% unique values of total values, data missingness (>30%), and multicollinearity (*r* > 0.80). After statistical screening, author knowledge again reduced the remaining >100 possible predictors to 69 probable predictors keeping in mind representing all four predictor domains under investigation and also to keep the predictor/patient ratio above 10 (12.96). To thereafter avoid human bias and achieve a robust final predictor set, algorithmic selection using recursive feature elimination (RFE) [32] was applied. At this step, RFE served as backwards stepwise predictor selection wrapper for an inner random forest classifier using all samples (*n* = 894) [33]. For the inner random forest classifier, the average Gini importance (reduction in node impurity) across CART decision trees in the random forest ensemble was used to rank predictors, meaning that a higher variable importance was assigned to predictors selected more often as root split predictors compared to predictors selected less often as root split or more often selected for descending leaf node splits. The RFE process is performed iteratively and it was here optimized on Accuracy starting with all predictors and then stepwise removing the least important predictors. To avoid overfitting this process, an outer wrapper of 3 × 7-fold cross-validation resampling was used. See pages 500–502 and Algorithm 19.5 in [34] for further details on how to control for overfitting the RFE predictor selection. The result defined the final predictor set thereafter used for model derivation and validation.

### Predictor types

See Table 1 below and Table S2 in the Appendix for detailed information on individual predictor variables. In summary, selected variables belong to different predictor types as exemplified: *Process* (time of day; day of week completing online MADRS-S screening). *Genetic* (PRSs for MDD, Autism Spectrum Disorder (ASD), Attention-Deficit Hyperactivity Disorder (ADHD), Bipolar Disorder (BPD), Intelligence (IQ), and Education (EDU)). Genotyping and PRS calculations have been described in detail in a previous publication [20] and references to the GWAS datasets used for PRS calculation are also in the Appendix (page 4). *Demographic* (age, sex, civic status, education, work experience). *Clinical* (symptom scales, medical history, psychotropic medication).

**Table 1:** Patient summary characteristics grouped by type and stratified by outcome.

	No Remission	Remission	Missing
*n*	451	338	105
Process			
ICBT start week of year	25.8 (16.1)	24.1 (15.6)	26
MADRS-S time of day	15:06:08 (5:10:04)	16:01:15 (4:71:03)	29
EQ5D time to complete	141.4 (308.2)	133.96 (362.1)	31
Genetic			
PRS-IQ (*p* ≤ 0.00001)	0.05 (0.99)	−0.01 (1.04)	0
PRS-IQ (*p* ≤ 0.001)	−0.07 (0.97)	0.08 (0.99)	0
PRS-IQ (*p* ≤ 0.05)	0.00 (1.00)	0.10 (0.96)	0
PRS-MDD (*p* ≤ 0.00001)	0.02 (0.98)	−0.06 (1.03)	0
PRS-MDD (*p* ≤ 0.05)	−0.04 (0.88)	−0.11 (0.88)	0
PRS-ASD (*p* ≤ 0.00001)	−0.00 (0.97)	0.03 (1.03)	0
PRS-ASD (*p* ≤ 0.001)	−0.00 (0.97)	0.02 (1.02)	0
PRS-ADHD (*p* ≤ 0.00001)	−0.08 (1.00)	0.04 (0.98)	0
PRS-ADHD (*p* ≤ 0.001)	−0.04 (0.97)	−0.09 (1.00)	0
PRS-BP (*p* ≤ 0.00001)	−0.07 (0.99)	0.06 (1.04)	0
PRS-EDU (*p* ≤ 0.001)	0.02 (0.95)	0.11 (0.99)	0
PRS-EDU (*p* ≤ 0.05)	0.07 (0.99)	0.08 (0.94)	0
PRS-Ancestry (*p* ≤ 0.001)	0.01 (1.07)	−0.02 (0.93)	0
Demographic			
Age	39.0 (11.9)	37.8 (11.7)	0
Education	4.9 (1.2)	5.2 (1.2)	2
Work experience	311 (69)	259 (77)	2
Clinical			
Prior mild MDD	57 (13)	59 (18.0)	35
Prior moderate MDD	96 (22)	72 (22)	35
Previous depression episodes	5.6 (7.9)	4.1 (5.2)	0
MADRS-S screen	26.4 (5.7)	23.3 (6.0)	0
MADRS-S pre	24.0 (5.8)	19.85 (6.0)	0
MADRS pre	21.8 (5.8)	19.7 (5.6)	47
PHQ-9 pre	16.6 (5.1)	13.7 (5.1)	29
EQ5D pre	0.6 (0.2)	0.7 (0.2)	5
EQ5D extreme anx/dep	186 (43)	89 (27)	31
EQ5D moderate pain	216 (50)	118 (36)	31
LSAS screen	45.0 (25.2)	38.1 (24.3)	88
PDSS screen	5.5 (5.6)	5.0 (5.4)	34
AUDIT screen	5.6 (4.9)	4.8 (4.4)	2
AUDIT-C screen	3.5 (2.1)	3.2 (2.0)	59
CGI-S pre	3.7 (0.7)	3.6 (0.8)	111
GSE pre	24.7 (4.9)	26.1 (5.0)	263
Prior psy meds	0.9 (1.2)	0.6 (0.9)	0
Current psy meds	1.3 (1.3)	0.9 (1.2)	0
No prior psy med	139 (32)	144 (44)	35
Any current psy med	235 (65)	147 (51)	157
Variable sleep-wake pattern	174 (51)	109 (43)	218
Reduced sex drive	181 (58)	131 (56)	272
Retarded speech	28 (8)	26 (10)	207
Reduced facial expressions	35 (10)	33 (13)	213
Agitation	39 (11)	27 (11)	208
GAF pre	61.1 (7.2)	62.7 (7.3)	214

Data are integer count (%) or decimal mean (SD). Total sample *n* = 894.

See Table S2 in the Appendix for a full description of variables.

*ADHD* attention-deficit hyperactivity disorder, *ASD* autism spectrum disorder, *AUDIT* Alcohol Use Disorders Identification Test, *BD* bipolar disorder, *CGIS* Clinical Global Impression Scale, *EQ5D-3L* EuroQoL’s quality of life five dimension scale with three level items, *EuroQoL* European Quality of Life group, *GAF* Global Assessment of Functioning, *GSS* General Self-efficacy Scale, *GWS* genome-wide significance, *ICBT* internet-delivered cognitive behaviour therapy, *LSAS* Liebowitz Social Anxiety Scale, *MADRS-S* Montgomery-Åsberg Depression Rating Scale-Self report, *MDD* major depressive disorder, *PDSS* Panic Disorder Severity Scale, *PRS* polygenic risk score, *SNP* single-nucleotide polymorphism.

### Data partitioning

To further control for overfitting in addition to resampling the predictor selection process and model training (internal validation), data were split into two datasets based on the date of patient treatment start. Thus, a training dataset used for model development (*n* = 537, 60% of cases) and a hold-out set for model validation included only the most recent patients (*n* = 357, 40%). Compared to a random split of data, a temporal non-random split is a stronger test of model generalisability since it provides a type of external validation [35, 36] through allowing for both random noise and temporal non-random bias to invalidate developed models.

### Model training

Models were trained using the same cross-validation as applied for feature selection. Although fairly balanced classes were present, downsampling of the majority class was used within each resampling fold to guarantee not overfitting to the majority class during model training [34]. Three algorithms of *deliberately increasing flexibility* were used to develop predictive models on the training dataset: (i) linear main-effects logistic regression with no tuning hyperparameters (LOGIT) [37], (ii) Breiman-type random forest with basic grid search tuning of the tree depth hyperparameter (RF) [38], and (iii) eXtreme Gradient Boosting machine which applies gradient descent to iteratively develop its individual trees, and here also tuned with Bayesian Optimization to better calibrate its joint optimal setting across several hyperparameters (XGB) [39]. Both (ii) and (iii) models automatically handle possible higher-order effects/interactions whereas model (i) requires manual specification of such terms and were deliberately not allowed to keep this model simple. Models (ii) and (iii) are ensemble algorithms that construct and combine a number of weak decision trees into a combined and usually more accurate model. Additionally, predicted probabilities obtained from model (i) to (iii) were also further combined into (iv) a blended meta-ensemble (META) model and compared versus the top performing individual model. Similar to how an RF model prediction of a sample is based on many internal individual model predictions (individual decision trees within the random forest), the META ensemble is a weighted combination of the separate model predictions from LOGIT, RF, and XBG. Obtaining the optimal blend of probabilities from the individual models involved using greedy optimization to iteratively tune individual model weights in the meta-ensemble towards *local optima* (best combination of model predictions versus a set of similar combinations) for each CV-resampling fold. Greedy optimization can thus fail to find the *global optima* (best combination of model predictions across all possible combinations) but is generally efficient at approximating the global optima within reasonable time (i.e., finding good individual model weights for an ensemble) [40].

### Model validation

Developed models were evaluated on the unseen hold-out validation set with retained real-world remission base rate (no downsampling) which are not perfectly balanced (44.5% in remission of total validation cases). Accuracy was the primary performance metric. We also report Area Under the receiver operating characteristics Curve (AUC, C-statistic) and positive/negative predictive values (PPV/NPV). Associated 95% confidence bounds (CI) are provided for both Accuracy and AUC. *P*-values for binomial tests of developed models versus the null model are reported, and AUC curves are also plotted against a random classifier. The null model is a no information model classifying all cases as belonging to the majority class (i.e., 55.5% correctly classified in the validation set). To test individual models against each other, their AUCs were bootstrapped (*n* bootstraps = 5000) and compared reporting the standard deviation of the difference between AUCs (D) and P for pairwise comparison.

### Model transparency analysis

Partial dependence plots for the two most important numeric predictors (as defined by RFE) for each of the four predictor types are provided and interpreted. Partial dependence shows the group-level influence of a single predictor on the probability for remission holding other predictors constant in the model. To further assess the influence of particular predictors on remission prediction in individual patients, the Local Interpretable Model-agnostic Explanations (LIME) procedure was applied [41]. This modelling involves several steps, but in summary, a ridge regression model (L2 penalization) was applied with the trained model on permutations (*n* = 5000) for each individual patient and the relative influence of top 10 predictors (largest model weights) for the probability of remission in six individual patients is presented. This detailed analysis of a few individuals is not representative of all patients yet does provide more insight into how single cases are classified by the non-linear model assuming local linearity.

### Additional statistics

Continuous variables are reported as arithmetic mean (SD) and categorical variables are reported as count (%) and further specified as needed. Missing values are reported descriptively and thereafter imputed with K-Nearest Neighbour imputation using the weighted Gower distance metric and k set low (*k* = 3) to preserve the correlational structure of data [42]. Analyses were performed in R [43] using packages *AppliedPredictiveModeling, caret, caretEnsemble, corrplot, data.table, doParallel, dplyr, dummies, foreign, ggplot2, ggpubr, ggthemes, haven, Hmisc, lattice, latticeExtra, lime, matrixStats, mice, mlbench, MLmetrics, pdp, pROC, randomForest, rBayesianOptimization, scales, stringr, tableone, VIM, vip*, and *xgboost*.

## Results

### Sample characteristics

In the full sample, observed pre-treatment MADRS-S total score was substantially higher (22.1 [6.3]) than at post-treatment (12.8 [5.8]) representing an almost halved (-42%) pre-post MADRS-S percentage change with 338 (43%) of patients in remission at the end of treatment.

Stratified by observed outcome, patients not in remission after treatment also scored higher (24.0 [5.8] vs 19.9 [6.0]) on pre-treatment MADRS-S and had a smaller percentage symptom decline (-22 [28] vs -68 [23]) pre-post treatment MADRS-S, compared to patients in post-treatment remission. Other outcome-stratified summary statistics are available in Table 1 showing that patients in remission started treatment a bit earlier in the year, completed the MADRS-S questionnaire slightly earlier during the day, and completed the EQ5D questionnaire faster, compared to patients not in remission. Moreover, remitted patients were marginally younger, had a higher education and more profession-specific work experience. Patients in remission also more commonly had a diagnostic history of mild MDD, fewer depressive episodes, scored lower on MDD severity, anxiety severity, and higher on quality of life and self-efficacy, reported less alcohol and medication use (both previous and present), and were overall rated more favourably on functionality by the interviewing psychiatrist.

### Predictor selection

Out of the 69 probable predictors, the RFE algorithm discarded 24 predictors and retained 45 predictors. This final predictor set included variables from all four predictor types (n Process= 3; Genetic= 13; Demographic= 3; Clinical= 26). See Table 1 for the final predictors set and **Appendix Figure S**1 for the RFE process.

### Predictor importance

**Fig. 2:**
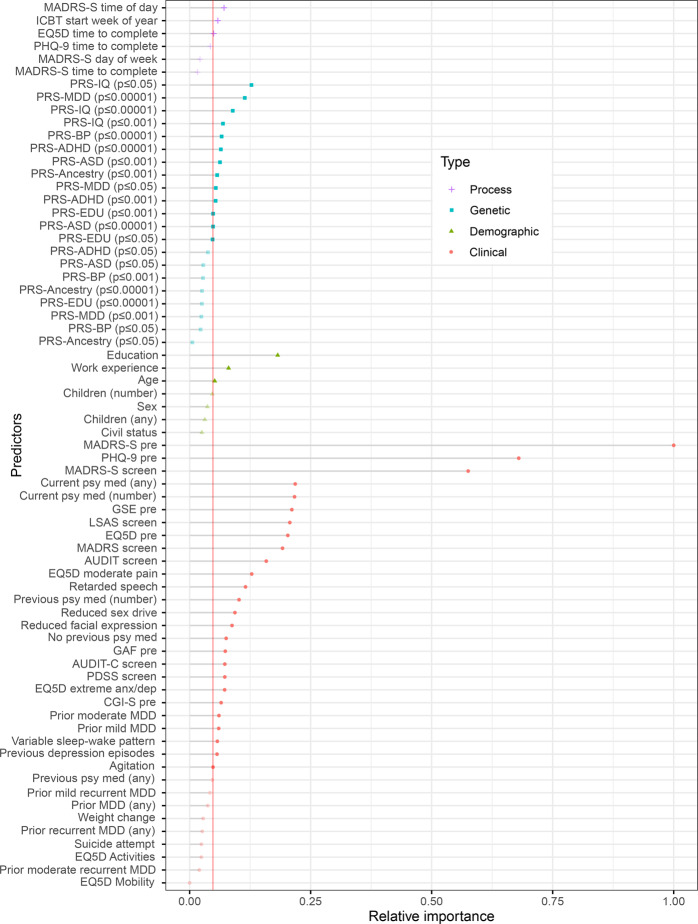
Individual predictors sorted by predictor type and relative Gini importance for predicting remission post ICBT in adults with MDD. Needle length on the x-axis represents relative importance with the strongest predictor scaled to 1 and others as proportional fractions of 1. Colour groups predictors into one of four categories (Process, Genetic, Demographic, and Clinical). The vertical line defines the RFE cut-off for final model inclusion which 45 retained variables (solid dot) and discarded 24 predictors (transparent dot). Total sample *n* = 894. *ICBT* Internet-based Cognitive Behaviour Therapy, *MDD* Major Depressive Disorder, *RFE* Recursive Feature Elimination.

The RFE result in Fig. 2 details the individual predictors sorted by their type and relative Gini importance (reduction in decision node impurity across trees). The most important Process predictor was time of day when the patient completed MADRS-S online, the most important Genetic predictor was the PRS for intelligence calculated with SNP GWS *p* ≤ 0.05. The most important Demographic predictor was educational attainment, and the most important Clinical predictor was pre-treatment total score MADRS-S. Overall, Clinical predictors dominated the other types with respect to both individual relative importance and total number selected by RFE. However, predictors of all types were ultimately included by the algorithm for classifying the post treatment remission target.

### Model validation

#### Individual model performance versus the null model

Predictive performance was quite similar across individual models. However, RF had the best performance (Accuracy [95%CI] p vs null model, PPV/NPV) on the hold-out test set outperforming the null model (0.656 [0.604, 0.705] 0.004, 0.573/0.731) which neither LOGIT (0.611 [0.558, 0.662] 0.181, 0.528/0.679) nor XGB (0.613 [0.561, 0.664] 0.154, 0.533/0.670) did.

#### Individual model performance versus one another

**Fig. 3:**
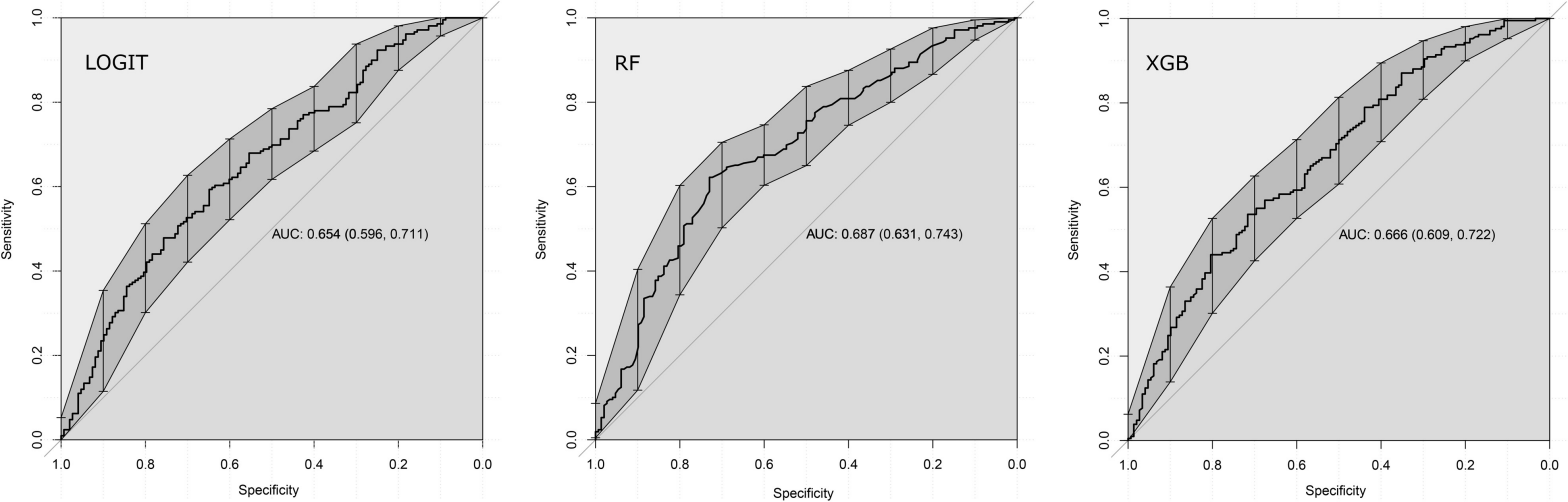
Individual model performance on the hold-out test set. *AUC* area under the receiver operating characteristics curve, *LOGIT* logistic regression, *RF* random forest, *XGB* extreme gradient boosted trees.

Pairing and bootstrap testing (*n* = 5000) of AUC curves on the hold-out validation set further showed only a statistical tendency of performance difference for LOGIT vs RF (D = 1.730, *p* = 0.084 favouring RF), and no significant performance difference comparing LOGIT vs XGB (D = 1.152, *p* = 0.249) or RF vs XGB (D = 0.463, *p* = 0.643). See Fig. 3 for further details.

#### Meta-ensembling

Correlations of individual models were suitable for meta-ensembling (predicted probabilities correlation range = 0.67–0.83) and a blend of LOGIT, RF and XGB was constructed. However, performance of META was not superior (*p* = 0.72) versus the single RF model and RF was accepted as the final model.

#### Cut-off sensitivity analysis

Re-classifying outcome with cut-off 12 (instead of 10) on the post ICBT MADRS-S rendered very similar performance of the RF model predicting reclassified patients (0.667 [0.615, 0.715], <0.001, 0.714/0.623) showing no statistical difference when bootstrapped and compared (D = -1.118, *p* = 0.264) suggesting that the original RF model is robust to slight variation in the cut-off used to define clinical remission.

#### Individual predictors

**Fig. 4:**
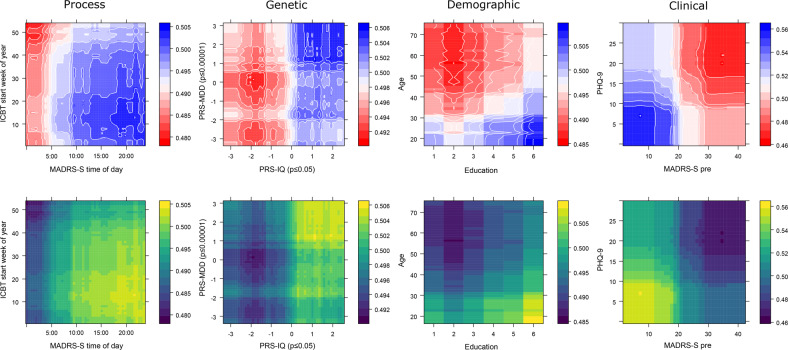
Partial prediction plots of the strongest two predictors by predictor type. Each column represents one of the four predictor types from which the two top numeric predictors have been plotted on their respective x and y axis. Colour represents the individual variable contribution to the predicted probability of remission in two ways. The upper panel row is colour-blindness friendly (Blue = higher probability of remission) and the bottom row is greyscale compatible (Light = higher probability of remission). The probability contribution of each variable in each panel is calculated when all other predictors in the model are centred and held constant.

Since the best performing model was a non-linear ensemble classifier, straightforward interpretation of model coefficients was impossible. Instead, partial dependence of most important numeric predictors sorted in pairs by predictor type are available in Fig. 4 showing individual predictor contributions to the probability of remission after ICBT when all other variables are mean centred and held constant. Graphs of individual predictors are presented in pairs (x and y axis) and by predictor type (column), with the probability for remission going from red (lower probability) to blue (higher probability) in the top row of facets. For Process predictors, we found that completing MADRS-S screening in the internet portal later in the day and starting treatment earlier in the year predicted remission. Regarding Genetics, having an intermediate or low PRS for depression predicted non-remission whereas a higher PRS for intelligence predicted remission. There was also some additive predictive power for remission having both a high PRS for depression and intelligence. With respect to Demographics, a lower age and higher education predicted remission. Considering Clinical predictors, having a low score on pre-treatment PHQ-9 and MADRS-S additively predicted remission.

#### Individual patient predictions

**Fig. 5:**
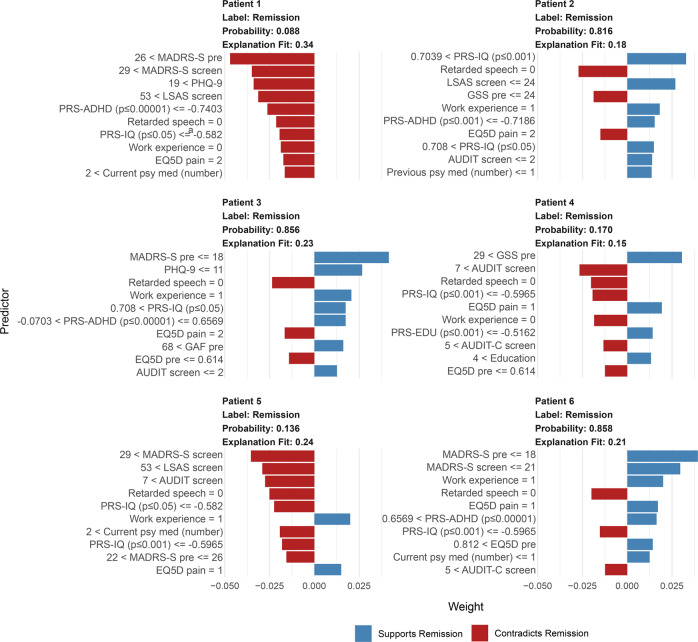
Individual case predictions exemplified with six individual patients. The top 10 predictors and their cut-off values influencing the probability for post ICBT remission in each patient. Data are from the final RF model with additional modelling using the LIME framework.

How predictors influenced the probability for post ICBT remission in six individual patients is available in Fig. 5 as LIME plots showing that the RF classifier reused several of the strongest predictors (e.g., MADRS-S, PRS for intelligence, latency of speech, PHQ-9) across patients. For example, a score below 19 indicated remission (Patient 3, Patient 6) but a score above 26 on MADRS-S contradicted remission (Patient 1, 2) and the pattern was similar for the other symptom scales. Also, a higher genetic PRS for intelligence indicated remission (Patient 2, 3) and lower contradicted remission (Patient 1, 4, 5, 6), a GSE score above 29 indicated remission (Patient 4). Having education-relevant professional experience indicated remission (Patient 2, 3, 5, 6) and vice versa (Patient 1, 4). Overall, scoring higher than 5 on AUDIT (alcohol), reporting substantial QoL-related physical pain, and taking more than one psychiatric medicine contradicted remission, whereas having a high education indicated remission. Finally, the model would classify Remission in Patient 2, 3, 6 and No Remission in Patient 1, 4, and 5.

## Discussion

The present study investigated a multi-modal prediction approach within a contemporary machine learning pipeline for predicting remission in a sample of adult patients with mild-to-moderate MDD that were genotyped and treated with state-of-the-art ICBT in routine psychiatric care in Sweden. Both at the group and individual patient level, the final model made use of all four of the investigated predictor types (process, genetic, demographic, and clinical). Although clinical predictors were strongest, PRSs for intelligence, depression, and other traits, ICBT process-specific variables, and demographic information, independently contributed to predicting remission. Modelling was (a) deliberately increased in complexity from log-linear main effects regression to non-linear and fine-tuned gradient boosting and meta-ensembling, (b) internally validated through robust resampling, and (c) externally validated on unseen, temporally separated data. Models were fairly accurate but with remaining room for improvement to reach usefulness as decision making support, suggesting a potential for future multi-modal prediction and implementation of such models for routine care prediction of post ICBT remission in patients with MDD.

Our findings corroborate previous findings of symptom severity, education, age, medication use, and PRSs as predictors for ICBT outcomes [3, 8, 10, 12, 13, 16, 20]. The number of predictors that our final model settled with was fairly high (*n* = 45), also consistent with the logic of a multi-modal approach for predicting complex traits and behaviours. Regarding remission of MDD in particular, our result is in line with Andersson and colleagues [11] in that depression symptom severity and higher anxiety predicted the outcome. Considering genetics, we only partially replicated the findings from a prior study [20] to the extent that a signal for the PRS for autism was found, although it was quite weak and surpassed by both the PRS for intelligence and PRS for MDD. The previous study used the same patients but it investigated symptom change over time using a linear mixed model, whereas the present study employed the use of multimodal data and machine learning for non-linear binary classification of remission post treatment. Somewhat surprisingly, a higher PRS for MDD was weakly positively associated with remission in partial dependence analysis whereas symptom severity at baseline was clearly negative for remission. One may possibly speculate that a genetic propensity for MDD may fit the treatment well, yet this goes against the pattern of higher PRS—higher symptom severity and less symptom improvement over time. Possibly instead, it may be that the association of lower MDD PRS—lower probability for remission would be driven by environmental factors. However, “factor” implies causality and the present design was not aimed at investigating that. Further, the present finding of higher PRS for intelligence—higher probability for remission seems reasonable given that ICBT is a highly verbal treatment, posing demands on the patient to not only be computer- and internet-proficient but also able to express fairly abstract concepts including internal emotions through an abstract medium (written text), and also to decide, plan, execute behavioural change. Having a high genetic propensity for intelligence in such a situation is likely beneficial for treatment success which would then feed into the probability of remission. We wish to highlight that the present study design is not optimized for investigating individual predictors, and although interesting and important to discuss from a hypothesis-generating viewpoint, the overall predictive power of specific PRSs were quite weak in the present study and interpretation of these and other specific predictors must be considered tentative and treated with appropriate caution.

The present study employed baseline pre-to-post prognostication. Previous studies have also used symptom change during early parts of ongoing ICBT to predict final outcome [18, 44] and a recent RCT suggests that such individual patient predictions can be of clinical benefit [45]. A main strength of within-treatment prediction is a probabilistic basis for adapting treatment while it is ongoing [18]. The strength of baseline prediction does however offset a weakness-by-design in within-treatment prediction, i.e., that baseline prediction provides the probabilistic basis for matching correct treatment to a patient before it is even initiated and thereby lowering the risk of termination, wasted resources, and unnecessary patient suffering. Future evidence may favour prediction from either baseline for guiding treatment matching or prediction within-treatment for adaptation. It is however likely that both approaches will be useful in a future, more data-driven psychiatry and today we view them as complementary.

### Clinical implications

The present verification of known Clinical and Demographic predictors strengthens these predictors and their potential place in routine care for adults with MDD undergoing ICBT. The identification of online Process predictors, as well as Genetic predictors suggests future possible pathways towards improved remission prediction in these patients. There are however important additional challenges when progressing from (a) identifying new *group-level* predictors for ICBT outcomes in MDD to (b) *individual patient-level* predictions that matter in clinical reality. Herein, we demonstrate how (a) → (b) modelling could be performed. More research is however needed to ascertain the extent by which these predictions could provide triage decision support for groups of patients and/or guide treatment choice for the individual patient. For example, a predicted low probability of being in remission after ICBT could inform the decision to offer alternative treatment before starting ICBT (face-to-face CBT, home-based CBT, medication).

The prediction performance of the best model herein was good although with considerable room for improvement before clinical implementation is pursued. A worthwhile next step would therefore be to extend the present model with a larger sample, and possibly also more predictors which would likely improve predictive power. Another extension would be to treat disequilibrium-pruned but thereafter unaggregated SNPs as a high-dimensional predictor space for a deep learning model—a class of algorithms known to perform particularly well with high-dimensional data [21]. Aggregate PRSs, as used herein, are by design very condense representations of genetic information and may thus have shed potentially predictive genetic information. With enough samples, a deep learning model should be able to use high-dimensional genetic input for predicting the target. Given the complex nature of MDD and remission after ICBT, phenotype registry predictors—which are uniquely available in national Swedish registries—such as birth complications and detailed medical history could also contribute additional predictive power. Another ambitious follow-up study would be a clinical trial including adults with MDD, preferably a superiority trial with randomization of clinicians to the prediction model as decision support for prediction-based tailored initial treatment choice versus treatment as usual. Effectiveness and cost-effectiveness could then be evaluated on both remission immediately after completed treatment and also on long-term outcomes of medication and healthcare utilisation, rehospitalisation, accidents, and more. Another important future study which demands relatively minor resources would be a test of the present model predictions against experienced clinicians predicting the same outcome as the model in a prospective sample of patients. Such a study design would ascertain model prediction accuracy directly relative to expert human benchmark performance. One may expect that experienced clinicians can accurately predict psychiatric outcomes yet empirical data suggest otherwise [46].

### Strengths and limitations

Strengths of the present study includes its high quality of measurements, and multi-modal prediction approach leveraging contemporary machine learning methodology with a real-world sample of genotyped and ICBT-treated patients with MDD. MDD is a polygenic trait influenced by a multitude of factors [14, 15]. Remission in MDD is in turn a complex behavioural outcome that is, for instance, influenced by evidence-based psychotherapy. Although a predictor is not necessarily predicated on causality, one may for complex multi-factorial phenomena expect a plethora of causal agents, and consequently, several diverse predictors. We cannot assume that our results predict outcome that is causally specific to ICBT treatment since the study was uncontrolled. It could be that precision psychiatry models have a general room for improvement insofar that sample sizes in the field tend to be relatively small, that non-clinical potential predictors are rarely investigated, and that there is an underutilisation of more flexible algorithms better able to detect subtly predictive patterns in complex data than linear main effect models are. However, model accuracy herein also showed substantial room for improvement. A larger sample would likely have helped in improving model predictive performance, suggesting for the future to both gather more samples and also to collaborate internationally with the few other sites with data of this rich multi-modal type available. The high genetic correlation across mood and anxiety disorders observed by others suggest that cross-disorder pooling of samples to gain modelling power would be worthwhile to pursue also for non-linear predictive modelling of post ICBT treatment outcome, in spite of some expected increased heterogeneity [17]. Regarding generalisability, the present model was developed with data from routine specialised care in Sweden and may not immediately generalise to other clinics, across different CBT treatments, beyond our national border, or to other patient groups. This is however also a strength because the high ecological validity means that results should be readily implementable at the nationwide recruiting IPSY clinic for patient treated with ICBT for mild-to-moderate MDD. Models often need to be retrained and weights adjusted to fit new contexts which could be accomplished given that samples are available from other clinics in Sweden or abroad. Patient online behaviour is logged automatically in every comprehensively designed ICBT platform, and from the log database one can extract and calculate Process variables that are rarely used as predictors for ICBT outcomes. This is potentially an underutilised source of predictive power for ICBT outcomes models and more research on this class of predictors seems warranted [12, 47].

## Conclusion

A new, multi-modal machine learning model for predicting MDD remission status after treatment with ICBT in routine care was derived and empirically validated. In future iterations, this model may inform tailored intervention before initiating ICBT for MDD. The multi-modal approach to predict remission in these patients was supported and warrants further investigation to establish clinical utility.

## Supplementary information

Appendix

## Data Availability

R code to generate results herein are available from the corresponding author upon reasonable request.
