# Supplementary material for: Predicting remission after internet-delivered psychotherapy in patients with depression using machine learning and multi-modal data
Source: Transl Psychiatry. 2022 Sep 1;12:357. doi: 10.1038/s41398-022-02133-3 (PMC9437007; doi:10.1038/s41398-022-02133-3)
Supplement: Supplementary file 1 — Appendix [file 41398_2022_2133_MOESM1_ESM.docx]

*Appendix for*

Predicting remission after internet-delivered psychotherapy in patients with depression using machine learning and multi-modal data

*by*

John Wallert, Julia Boberg, Viktor Kaldo, David Mataix-Cols, Oskar Flygare, James Crowley, Matthew Halvorsen, Fehmi Ben Abdesslem, Magnus Boman, Evelyn Andersson, and Christian Rück

CONTENT

1. Final predictor set………………………………………………………………………………….p2

2. RFE selection process……….……………………………………………………………………..p4

2. GWAS data for calculating PRSs………………………………………………………………….p4

3. Appendix references…………………………………………………………………………….....p5

**1. Final predictor set**

| **Table S2. Additional information on the final predictors sorted by type** | | |
| --- | --- | --- |
| **PREDICTOR** | **DESCRIPTION** | **VALUES** |
| **Process** |  |  |
| ICBT start week of year | Week of year that a patient began ICBT treatment | 1 – 52 |
| MADRS-S time of day | Time during the day (HH:MM:SS) that a patient completed the pre-treatment MADRS-S questionnaire online in the portal | 00:03:38 – 23:59:59 |
| EQ5D time to complete | Time in seconds that a patient took to complete the pre-treatment EuroQoL questionnaire online in the portal | 12 – 5243 |
| **Genetic** |  |  |
| PRS-IQ (p≤0.00001) | Polygenic Risk Score for Intelligence with SNP threshold for score inclusion at genome-wide significance p≤0.00001 | -2.94263 – 3.67183 |
| PRS-IQ (p≤0.001) | Polygenic Risk Score for Intelligence with SNP threshold for score inclusion at genome-wide significance p≤0.001 | -3.56201 – 2.51096 |
| PRS-IQ (p≤0.05) | Polygenic Risk Score for Intelligence with SNP threshold for score inclusion at genome-wide significance p≤0.05 | -3.28852 – 3.06275 |
| PRS-MDD (p≤0.00001) | Polygenic Risk Score for Major Depressive Disorder with SNP threshold for score inclusion at genome-wide significance p≤0.00001 | -3.31445 – 3.06800 |
| PRS-MDD (p≤0.05) | Polygenic Risk Score for Major Depressive Disorder with SNP threshold for score inclusion at genome-wide significance p≤0.05 | -2.96219 – 2.63801 |
| PRS-ASD (p≤0.00001) | Polygenic Risk Score for Autism Spectrum Disorder with SNP threshold for score inclusion at genome-wide significance p≤0.00001 | -3.06872 – 3.01786 |
| PRS-ASD (p≤0.001) | Polygenic Risk Score for Autism Spectrum Disorder with SNP threshold for score inclusion at genome-wide significance p≤0.001 | -2.97747 – 4.37585 |
| PRS-ADHD (p≤0.00001) | Polygenic Risk Score for Attention-Deficit Hyperactivity Disorder with SNP threshold for score inclusion at genome-wide significance p≤0.00001 | -3.50762 – 3.59199 |
| PRS-ADHD (p≤0.001) | Polygenic Risk Score for Attention-Deficit Hyperactivity Disorder with SNP threshold for score inclusion at genome-wide significance p≤0.001 | -2.85362 – 3.24018 |
| PRS-BP (p≤0.00001) | Polygenic Risk Score for Bipolar Disorder with SNP threshold for score inclusion at genome-wide significance p≤0.00001 | -2.78376 – 3.04073 |
| PRS-EDU (p≤0.001) | Polygenic Risk Score for Educational attainment with SNP threshold for score inclusion at genome-wide significance p≤0.001 | -3.00270 – 2.81852 |
| PRS-EDU (p≤0.05) | Polygenic Risk Score for Educational attainment with SNP threshold for score inclusion at genome-wide significance p≤0.05 | -3.34865 – 2.88809 |
| PRS-Ancestry | Ancestry loading | -3.03424 – 5.53610 |
| **Demographic** |  |  |
| Age | Patient age at baseline | 18 – 75 |
| Education | Patient educational attainment at screening | 1 – 6 |
| Work experience | Patient education-relevant professional experience | Yes, No |
| **Clinical** |  |  |
| Prior mild MDD | Prior diagnosis of MDD of mild severity | Yes, No |
| Prior moderate MDD | Prior diagnosis of MDD of moderate severity | Yes, No |
| Previous depression episodes | Number of previous depressive episodes | 0 – 4 |
| MADRS-S screen | Montgomery-Åsberg Depression Rating Scale-Self report total score at screening | 4 – 44 |
| MADRS-S pre | Montgomery-Åsberg Depression Rating Scale-Self report total score at pre-treatment | 2 – 42 |
| MADRS pre | Montgomery-Åsberg Depression Rating Scale total score at pre-treatment (clinician rated). | 1 – 39 |
| PHQ-9 pre | Patient Health Questionnaire total score at pre-treatment | 0 – 29 |
| EQ5D pre | EuroQol’s EQ5D total index score | -0.1350 – 1.0000 |
| EQ5D extreme anx/dep | Patient reporting as extremely anxious or depressed on this EQ5D-3L single item | Yes, No |
| EQ5D moderate pain | Patient reporting moderate physical pain on this EQ5D-3L single item | Yes, No |
| LSAS screen | Liebowitz Social Anxiety Scale total score at screening | 0 – 141 |
| PDSS screen | Panic Disorder Severity Scale total score at screening | 0 – 23 |
| AUDIT screen | Alcohol Use Disorders Identification Test, full scale total score at screening | 0 – 32 |
| AUDIT-C screen | Alcohol Use Disorders Identification Test-Consumption, consumption subpart of the scale total score at screening | 0 – 11 |
| CGI-S pre | Clinical Global Impression Severity scale score at pre-treatment | 2 – 6 |
| GSE pre | General Self-Efficacy Scale total score at pre-treatment | 10 – 40 |
| GAF pre | Global Assessment of Functioning Scale total score pre-treatment |  |
| Prior psy meds | Number of psychotropic medications used previously | 0 – 5 |
| Current psy meds | Number of psychotropic medications currently used | 0 – 5 |
| No prior psy med | No previous psychotropic medication intake | Yes, No |
| Any current psy med | Any currently used psychotropic medication | Yes, No |
| Variable sleep-wake pattern | Melancholia assessment: variable diurnal sleep-wake pattern | Yes, No |
| Reduced sex drive | Melancholia assessment: reduced sex drive | Yes, No |
| Retarded speech | Melancholia assessment: retarded speech | Yes, No |
| Reduced facial expressions | Melancholia assessment: reduced facial muscular activity | Yes, No |
| Agitation | Melancholia assessment: agitation | Yes, No |
| Total sample n= 894. | | |

**2. RFE predictor selection process**


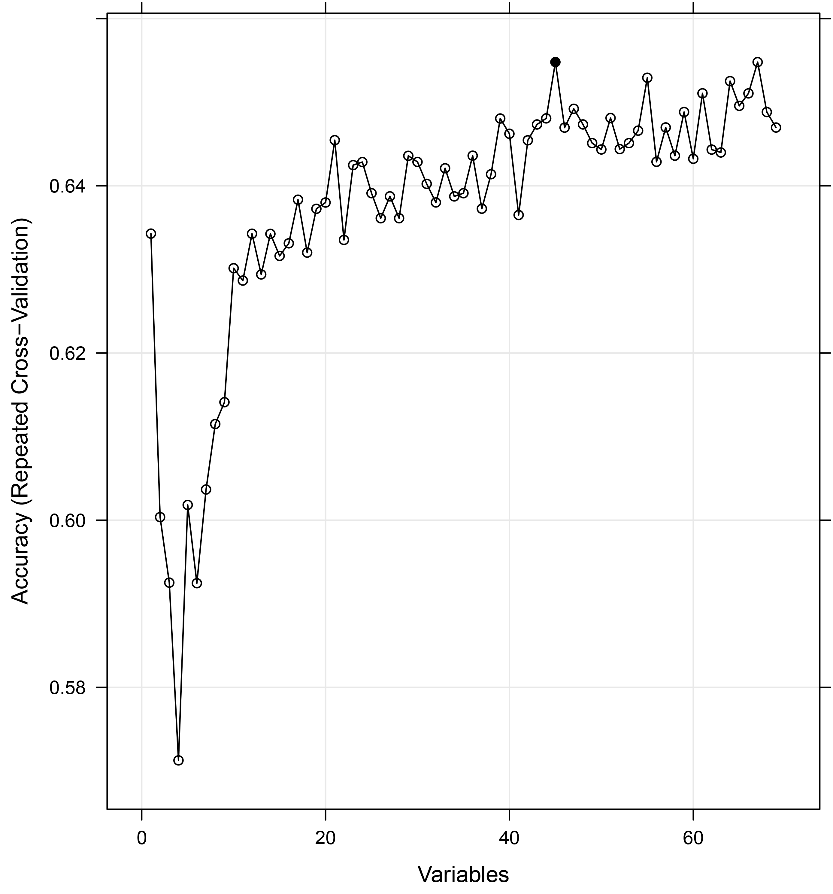


**Figure S1. Recursive feature elimination (RFE) with resampling.** Dots represent the resampled point estimate accuracy for predicting post ICBT remission at each number of evaluated predictors. The single filled dot represents the RFE decision of optimal n predictors to include in the final model. Starting with all 69 predictors variables as input (right hand side), the RFE process iteratively removed predictors until it ultimately retained 45 final predictors for the final predictor set. Predictor ranking determined which predictors to include in the final model. Resampling is an outer 3x7-fold repeated cross-validation to avoid overfitting.

**3. GWAS datasets for calculating PRSs**

The methodology for PRS calculation follows standard practices and is detailed on pages 486-487 in (Andersson et al., 2019). GWAS datasets used for PRS calculation were provided by the Psychiatric Genomics Consortium (PGC) website (<https://www.med.unc.edu/pgc/download-results/>) and the following were used for Major Depressive Disorder,(Wray et al., 2018) Bipolar Disorder,(Stahl et al., 2019) Attention-Deficit/Hyperactivity Disorder,(Demontis et al., 2019) and Autism Spectrum Disorder,(Anney, Ripke, Verneri, & the Autism Spectrum Disorders Working Group of The Psychiatric Genomics Consortium, 2017). For Intelligence, as well as Educational attainment, other published GWAS meta-analyses were used as input data for PRS calculation.(Okbay et al., 2016; Sniekers et al., 2017).

**4. Appendix references**

Andersson, E., Crowley, J. J., Lindefors, N., Ljotsson, B., Hedman-Lagerlof, E., Boberg, J., . . . Ruck, C. (2019). Genetics of response to cognitive behavior therapy in adults with major depression: a preliminary report. *Mol Psychiatry, 24*(4), 484-490. doi:10.1038/s41380-018-0289-9

Anney, R. J. L., Ripke, S., Verneri, A., & the Autism Spectrum Disorders Working Group of The Psychiatric Genomics Consortium. (2017). Meta-analysis of GWAS of over 16,000 individuals with autism spectrum disorder highlights a novel locus at 10q24.32 and a significant overlap with schizophrenia. *Mol Autism, 8*, 21. doi:10.1186/s13229-017-0137-9

Demontis, D., Walters, R. K., Martin, J., Mattheisen, M., Als, T. D., Agerbo, E., . . . Neale, B. M. (2019). Discovery of the first genome-wide significant risk loci for attention deficit/hyperactivity disorder. *Nat Genet, 51*(1), 63-75. doi:10.1038/s41588-018-0269-7

Okbay, A., Beauchamp, J. P., Fontana, M. A., Lee, J. J., Pers, T. H., Rietveld, C. A., . . . Benjamin, D. J. (2016). Genome-wide association study identifies 74 loci associated with educational attainment. *Nature, 533*(7604), 539-542. doi:10.1038/nature17671

Sniekers, S., Stringer, S., Watanabe, K., Jansen, P. R., Coleman, J. R. I., Krapohl, E., . . . Posthuma, D. (2017). Genome-wide association meta-analysis of 78,308 individuals identifies new loci and genes influencing human intelligence. *Nat Genet, 49*(7), 1107-1112. doi:10.1038/ng.3869

Stahl, E. A., Breen, G., Forstner, A. J., McQuillin, A., Ripke, S., Trubetskoy, V., . . . & the Bipolar Disorder Working Group of the Psychiatric Genomics Consortium. (2019). Genome-wide association study identifies 30 loci associated with bipolar disorder. *Nat Genet, 51*(5), 793-803. doi:10.1038/s41588-019-0397-8

Wray, N. R., Ripke, S., Mattheisen, M., Trzaskowski, M., Byrne, E. M., Abdellaoui, A., . . . Major Depressive Disorder Working Group of the Psychiatric Genomics, C. (2018). Genome-wide association analyses identify 44 risk variants and refine the genetic architecture of major depression. *Nat Genet, 50*(5), 668-681. doi:10.1038/s41588-018-0090-3
